# Age determines response to anti-TNFα treatment in patients with ankylosing spondylitis and is related to TNFα-producing CD8 cells

**DOI:** 10.1007/s10067-018-4061-y

**Published:** 2018-03-15

**Authors:** Agata Schramm-Luc, Jolanta Schramm, Mateusz Siedliński, Tomasz J. Guzik, Bogdan Batko

**Affiliations:** 1 Department of Internal and Agricultural Medicine, Faculty of Medicine, Medical College, Jagiellonian University, Cracow, Poland; 2 Department of Rheumatology, J. Dietl Specialist Hospital, ul. Skarbowa 1, 31-121 Cracow, Poland

**Keywords:** Activation markers, Ageing, Clinical improvement, Cytokines, Predictors, TNFα inhibitors

## Abstract

Younger age is a predictor of good clinical response to treatment with tumour necrosis factor (TNF) α inhibitors in ankylosing spondylitis (AS) patients; therefore, the aim of the study was to determine age-related differences in cellular functions, which can predict the response. High disease activity AS patients were treated with TNFα inhibitors for 12 weeks. Based on the percentage of Bath Ankylosing Spondylitis Disease Activity Index (BASDAI) improvement, patients were divided into responding or non-responding groups. Cytometric and clinical assessment were determined at baseline, 4, and 12 weeks after initiation of anti-TNFα treatment. Expression of activation markers on T cells and intracellular cytokine staining was performed. Baseline percentage of TNFα-producing CD8 cells was lower in responders than in non-responders (20.8 ± 2.9 vs 40.7 ± 8.2; *P* = 0.04 in *T* test) and increased in the responding group during the first month of treatment (20.8 ± 2.9 vs 30.3 ± 2.5; *P* = 0.02). Moreover, its baseline level correlated with age (*r* = 0.7; *P* = 0.0009) but not with BASDAI improvement adjusted for age. There were no differences in the baseline percentage of IL-4, IL-17A, and IFNγ within CD4 and CD8 cells nor in the expression of CD25, CD28, and CD69 on these cells between responders and non-responders. However, baseline level of CD4+CD28null cells correlated with the percentage of BASDAI improvement while analysed as a continuous variable adjusted for age (*r* = − 0.4; *P* = 0.048). Clinical predictors of response were also determined. Influence of age on the response to anti-TNFα treatment in AS patients could be mediated by TNFα-producing CD8 cells.

**Electronic supplementary material:**

The online version of this article (10.1007/s10067-018-4061-y) contains supplementary material, which is available to authorized users.

## Introduction

Ankylosing spondylitis (AS) is an inflammatory spondyloarthropathy more prevalent in younger age, often impairing life quality when treatment is not successful. In spite of numerous studies, the number of drugs which can be used for effective treatment of AS is limited. Conventional synthetic disease-modifying antirheumatic drugs (csDMARDs) have proven ineffective in treatment of the axial form of AS [1, 2]. Biological agents are considered the next line of treatment, in case of non-steroidal anti-inflammatory drug (NSAID) inefficiency [1].

Tumour necrosis factor (TNF) α is one of the principal cytokines involved in the pathogenesis of inflammatory spondyloarthropathies, and drugs affecting this pathway are used in patients with high disease activity when other treatment options have proven ineffective. Currently, the decision to introduce anti-TNFα treatment is evaluated based on high disease activity, in clinical practice usually assessed using the Bath Ankylosing Spondylitis Disease Activity Index (BASDAI), and a lack of improvement in previous treatment. A large percentage of patients treated with TNFα inhibitors achieve major improvement; however, there is a significant percentage of patients who either do not respond to this treatment or in whom the response is lower than expected [3–5].

Several studies report that younger age is a predictor of good clinical response; however, it is unclear how age affects response to anti-TNFα treatment [3–5]. The aim of our study was to determine whether there are any age-related differences in cellular function, which can predict response to anti-TNFα treatment in AS patients.

In AS patients who do not respond to anti-TNFα treatment, as a second line treatment, anti-IL-17 drugs are recommended, along with switching to another anti-TNFα agent [1]. Therefore, in this study, particular interest was given to the baseline level of IL-17 and its influence on clinical response.

## Materials and methods

### Study group

Twenty-four patients with AS, diagnosed based on the modified New York criteria [6], were involved in the study. All the patients were anti-TNFα naïve and were characterised by high disease activity ≥ 4 assessed using the Bath Ankylosing Spondylitis Disease Activity Index (BASDAI) [7] twice prior to the study with a 12-week interval. Patients were also shown to have an ineffective response to treatment with two non-steroidal anti-inflammatory drugs (NSAIDs) administered consecutively for 3 months each in the maximal recommended or tolerated dose, in accordance with reimbursement requirements. Patients were consecutively recruited into the study.

Patients with a history of hepatitis, pneumonia, pyelonephritis within the last 3 months, opportunistic infection within the last 2 months, joint infection within the last year, neoplasm within the last 5 years, or pre-cancer stage were excluded from the study.

All patients were receiving anti-TNFα agents in the standard dose for 12 weeks. Pre- and post-treatment disease activity was assessed, and based on the percentage of BASDAI improvement, patients were divided into two groups. Patients with a BASDAI improvement of 50% or greater (BASDAI 50) were classified as responders, while patients with an improvement lower than 50% were classified as non-responders. These two groups were further compared to search for predictors of good clinical response.

Blood sample collection and clinical assessment were performed at baseline, 4, and 12 weeks after starting anti-TNFα treatment.

This study is in accordance with ethical principles of the Helsinki declaration. The investigation protocol was approved by the Bioethics Committee of the Regional Chamber of Physicians in Cracow, Poland—investigation number 77/KBL/OIL/2013. Prior to enrolment, written consent was obtained from all patients.

### Assessment of the disease activity

Disease activity was assessed using BASDAI, patient global assessment on the visual analogue scale (VAS), and numerical rating scale for back pain, peripheral arthritis, and morning stiffness. Ankylosing Spondylitis Disease Activity Score (ASDAS) using the C-reactive protein (CRP) level was also calculated [8]. CRP was assessed using immunoturbidimetric test.

### Blood sample collection

Blood samples were collected from 20 patients into tubes containing ethylenediaminetetraacetic acid (EDTA). Plasma was separated by whole blood centrifuging. To isolate peripheral blood mononuclear cells (PBMCs), standard gradient centrifugation on Lymphocyte Separation Medium (LSM) 1077 (PAA Laboratories GmbH, Austria) was performed.

### Staining protocol

After isolation, cells were washed twice with a buffer composed of phosphate-buffered saline (PBS) and 1% heat-inactivated fetal bovine serum (FBS) from Gibco (Life Technologies, USA) and suspended within. Cell count was evaluated by Fuchs-Rosenthal chamber. Next, 5 × 10^5^ PBMCs were stained with monoclonal antibodies: anti-CD3–PerCP (Clone SK7), anti-CD4–PE-Cy7 (Clone SK3), anti-CD8–APC-H7 (Clone SK1), anti-CD25–PE (Clone M-A251), anti-CD69–FITC (Clone FN50), anti-CD28–APC (Clone CD28.2) from BD Biosciences (San Jose, CA, USA). Cells were stained at a temperature of 4 °C in the dark for 20 min, then washed with buffer. Next, cells were suspended in 200 μl of PBS+1%FBS and analysed in FACSVerse flow cytometer from BD Biosciences (San Jose, CA, USA). Collected data were further analysed in Flow Jo v10 (Ashland, OR, USA). Forward-scattered and side-scattered light (FSC/SSC) was used to separate lymphocytes. Within this population, T cells were separated based on the presence of CD3 marker. Next, among this population, two subpopulations were determined: CD4 and CD8 cells, and on each of them, presence of activation markers was investigated. Expression of the CD28 marker was defined using dot-plots, while for CD25 and CD69 markers, cut-off points were settled on Fluorescence Minus One (FMO) histograms.

### Assessment of intracellular cytokines

10^6^ PBMCs were suspended in RPMI 1600 medium (Gibco, Life Technologies, USA) with 10% FBS and 200 mM l-glutamine and 5 mg/ml gentamicin (Sigma-Aldrich, Saint Louis, MO, USA). Cells were stimulated with Leukocyte Activation Cocktail with BD GolgiPlug from BD Biosciences (San Jose, CA, USA) containing phorbol ester, PMA (phorbol 12-myristate 13-acetate), a calcium ionophore (ionomycin), and brefeldin A and cultured in a 5% CO_2_ humidified atmosphere in a temperature of 37 °C for 4 h, then washed with PBS+1%FBS buffer. Next, to determine superficial markers, cells were stained with monoclonal antibodies: anti-CD3–PerCP (Clone SK7), anti-CD4–APC (Clone RPA-T4), anti-CD8–APC-H7 (Clone SK1) from BD Biosciences and washed again with PBS+1%FBS. Permeabilisation solution (BD Biosciences) was added to the cells, then after 20 min, cells were washed with Perm Wash/Buffer (BD Biosciences). Next, using the following monoclonal antibodies: anti-IFNγ–FITC (Clone B27), anti-IL-17A-PE (Clone N49–653), anti-TNF–FITC (Clone MAb11), and anti-IL-4–PE (Clone 8D4–8), intracellular staining was performed, after which cells were washed with Perm/Wash Buffer. These prepared cells were suspended in PBS+1%FBS and collected using a FACSVerse flow cytometer from BD Biosciences (San Jose, CA, USA). In some cases, isotype controls IgG_1_ κ–FITC (Clone MOPC-21) for IFNγ and TNF and IgG_1_κ–PE (Clone MOPC-21) for IL-17A and IL-4 were used. Staining and permeabilisation were performed for 20 min in the dark at 4 °C.

### Statistics

Normality of distribution of all analysed variables was tested using the Shapiro-Wilk test for analysed groups, and if necessary, normality was achieved by log transformation. Correlation between BASDAI improvement and clinical characteristics was determined using the Pearson correlation, *T* test, Mann-Whitney *U* test, or chi-square test. *T* test was performed to test for differences in cell subpopulations between responding and non-responding groups. BASDAI improvement was also analysed as a continuous variable after adjustment for age, i.e., as residuals from linear regression with BASDAI improvement set as a dependent variable and age set as an independent variable. *P* values < 0.05 were considered significant. All tests were performed in IBM SPSS Statistics (ver. 23).

#### Data availability

The authors declare that they have full control of all primary data and that they agree to allow the journal to review their data if requested.

## Results

### Clinical and demographic variable

After 12 weeks of anti-TNFα treatment, in 13 (54.2%) out of 24 patients, BASDAI improved by 50% or more, while in 11 (45.8%) subjects, there was no response or the response was suboptimal. These two groups were further compared for predictors of good clinical response.

A significant difference in patients’ age was observed—mean age of the responding group was 13 years lower than the non-responding group. Groups also differed by the age at which disease was diagnosed, but not by duration of the disease (Table 1). There was a negative correlation between the percentage of BASDAI improvement and patients’ age (*r* = −0.6; *P* = 0.005).

**Table 1:** Baseline clinical characteristics of patients responding and not responding to anti-TNFα treatment

	Responding group (*n* = 13)	Non-responding group (*n* = 11)	*T* test *p* value
Demographic factors			
Age [years]	31.3 ± 5.8	44.4 ± 11.2	0.001
Sex	M 10 (76.9%) F 3 (23.1%)	M 9 (81.8%) F 2 (18.2%)	0.8
BMI—baseline	28.3 ± 3.5	26.7 ± 3.6	0.3
Body mass—baseline [kg]	83.9 ± 11.0	79.6 ± 15.4	0.4
Current smoking	4 (30.8%)	3 (27.3%)	0.9
Disease-related factors			
Duration of the disease [years]	6.4 ± 4.4	7.7 ± 8.5	0.7
Age at diagnosis	24.9 ± 7.6	36.6 ± 11.1	0.006
HLA-B27	11 (84.6%)	10 (90.9%)	0.6
BASDAI	6.9 ± 1.1	7.4 ± 0.9	0.2
ASDAS-CRP	4.1 ± 0.7	4.0 ± 0.6	0.6
CRP [mg/l]	21.0 ± 18.3	16.8 ± 17.8	0.3
CRP > 10 mg/l	10 (76.9%)	5 (45.5%)	0.1
VAS pain	71.2 ± 14.7	66.3 ± 10.1	0.4
Back pain^a^	8.0 (7.0–8.0)	8.0 (7.5–8.0)	0.3
Peripheral arthritis^a^	7.0 (4.0–7.0)	8.0 (7.0–8.0)	0.04
Morning stiffness^a^	7.0 (6.0–9.0)	7.0 (4.0–8.5)	0.6
Drugs			
Anti-TNFα			0.9
• Adalimumab • Etanercept • Infliximab	4 (30.8%) 7 (53.8%) 2 (15.4%)	3 (27.3%) 7 (63.6%) 1 (9.1%)	
csDMARDs total	4 (30.8%)	5 (45.5%)	0.5
• Methotrexate • Sulfasalazine	2 (15.4%) 2 (15.4%)	2 (18.2%) 3 (27.3%)	0.7

Sex, current smoking, HLA-B27, CRP > 10 mg/l, and drugs are shown as numbers of patients, others as mean ± SD

^a^For numeric data, such as back pain, peripheral arthritis, and morning stiffness, the Mann-Whitney *U* test was performed. Data are shown as median and IQR

There were no significant differences in sex, BMI, disease duration, presence of HLA-B27, baseline CRP, VAS pain, ASDAS-CRP, or BASDAI between these groups. However, groups differed by baseline peripheral arthritis, which was lower in the responding group. Number of patients receiving DMARDs or types of anti-TNFα drug did not differ between these groups (Table 1).

### T cell subsets

First, percentages of T cell subsets in both groups were compared. There was no difference in percentages of CD4 and CD8 cells between the responding and non-responding groups.

### Intracellular cytokine staining

**Fig. 1:**
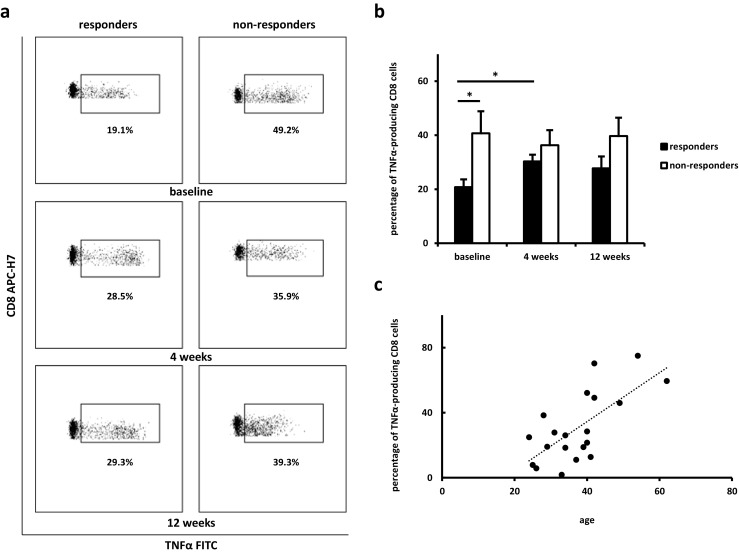
Differences in the intracellular TNFα staining of CD8 cells. Cytometric examples (**a**) and changes in the percentages of cells (**b**) in responding and non-responding groups at baseline, after 4, and 12 weeks from introducing anti-TNFα treatment. Data are shown as mean ± SEM; *P < 0.05. **c** Correlation between baseline percentage of TNFα-producing CD8 cells and patients’ age (*r* = 0.7; *P* = 0.0009)

Intracellular staining of IL-4, TNF, IFNγ, and IL-17A within CD4 and CD8 cells was performed. At baseline, a significant difference in the percentage of TNFα-producing CD8 cells between responding and non-responding groups was observed (Table 2, Fig. 1a, b) and baseline level of this cell subset significantly correlated with age (*r* = 0.7; *P* = 0.0009) (Fig. 1c).

**Table 2:** Baseline cytometric characteristic of patients responding and not responding to anti-TNFα treatment

	Responding group (*n* = 10)	Non-responding group (*n* = 10)	Non-paired *T* test *p* value
Intracellular cytokines			
CD4 IL-17A	0.2 ± 0.05	0.2 ± 0.05	0.98
CD4 IL-4	0.2 ± 0.07	0.5 ± 0.1	0.08
CD4 IFNγ	6.6 ± 1.4	6.3 ± 1.3	0.9
CD4 TNFα	26.9 ± 4.1	32.0 ± 6.1	0.5
CD8 IFNγ	19.5 ± 2.5	26.7 ± 5.4	0.3
CD8 TNFα	20.8 ± 2.9	40.7 ± 8.2	0.04
Activation markers			
CD4 CD28 null	2.6 ± 1.0	5.5 ± 2.3	0.5
CD4 CD25	38.8 ± 1.5	38.1 ± 4.2	0.6
CD4 CD69	4.3 ± 1.5	2.0 ± 0.6	0.2
CD8 CD28 null	26.0 ± 4.1	36.3 ± 6.5	0.2
CD8 CD25	7.5 ± 1.2	13.8 ± 4.8	0.6
CD8 CD69	5.5 ± 1.7	4.3 ± 1.8	0.7

Data are shown as mean percentage of cells ± SEM

Changes in this cell subset during anti-TNFα treatment were determined. After 12 weeks, there was no significant difference in the percentage of CD8+TNFα+ cells between responding and non-responding groups. Moreover, the percentage of TNFα-producing CD8 cells significantly increased in the responding group during the first month of treatment (Fig. 1a, b).

There was no difference between responding and non-responding groups in the baseline percentage of IL-4, IL-17A, and IFNγ within CD4 and CD8 cells, nor TNFα-producing CD4 cells (Table 2). Similarly, none of them correlated with BASDAI improvement after adjustment for age.

### Activation markers

Expression of CD25, CD28, and CD69 was determined on CD4 and CD8 cells. There were no differences in the expression of the activation markers on these T cell subsets between responding and non-responding groups. However, while analysing as a continuous variable, and after age adjustment, baseline level of CD4+CD28null cells negatively correlated with the percentage of BASDAI improvement (*r* = − 0.4; *P* = 0.048).

## Discussion

Despite numerous studies, it is unclear why some AS patients achieve significant improvement with anti-TNFα treatment, while others do not respond to the same treatment as expected. Although older age is a predictor of poor clinical response in AS patients, senescence of the immune system is a complex process influenced not only by patients’ age but also many other factors resulting in chronic low-grade inflammation [9]. Chronic stimulation of the immune system results in increased cell proliferation, reprogramming, and ageing [10]. Identification of mechanisms through which age affects cellular function and response to the anti-TNFα treatment will be an important step towards personalisation of the therapy.

In this study, we compared baseline features of AS patients responding or not responding to anti-TNFα treatment after 12 weeks. Similar to other studies, more than half of these patients reached BASDAI 50 after 12 weeks, and the responding group was significantly younger than the non-responding group [3–5]. Correspondingly, in our study, age negatively correlated with the percentage of BASDAI improvement.

Further comparison revealed that the percentage of TNFα-producing CD8 cells was significantly lower in the responding group than in the non-responding group and this cell subset correlated with age. In BASDAI improvement analysis, adjusted for age, there was no relationship with the percentages of these cells. This allows us to presume that the differences in the percentage of TNFα-producing CD8 cells between responding and non-responding group are caused by age. Previous studies reported increased concentration of TNFα in plasma [11] and within CD8 cells in an elderly population, particularly in memory and effector/cytotoxic subsets [12].

Furthermore, in our study, negative correlation between baseline percentage of CD4+CD28null cells and age-corrected BASDAI improvement was observed. This finding corresponds with increased intracellular staining of TNFα since its production is more frequent in CD28null than in CD28+ cells [13]. In rheumatoid arthritis (RA) patients, culturing of CD4+CD28+ T cells with TNFα results in the reduction of CD28 expression on CD4 cells and generation of the CD4+CD28null subset by influencing CD28 gene transcription [14]. Although this cell subset increases with age, mostly during the sixth to seventh decade of life [15], we did not observe this correlation. This can be explained by the fact that the percentage of CD4+CD28null cells is higher in AS patients than in age-matched healthy controls and correlates with disease status rather than a patient’s chronological age [16]. Moreover, the majority of patients recruited into our study were younger, which could explain the lack of additive effect of age.

We did not observe any influence of baseline expression of CD25 and CD69 markers on the response to anti-TNFα treatment. Although a high level of CD4+CD25+ is a predictor of response to infliximab in RA patients [17], results cannot be transferred to AS patients. Anti-TNFα treatment results in a decrease of CD4+CD25+ cells in AS patients [18]; however, in RA patients, it restores CD4+ CD25+ cells to the level of healthy controls [19], while in patients with inflammatory bowel disease, it has no effect on blood levels of these cells [20]. Responders and non-responders in our study did not differ in the baseline percentage of IL-17-producing CD4. Similarly, AS patients responding to or not responding to anti-TNFα treatment according to ASAS criteria did not differ in baseline levels of CD4+CD25+FoxP3+ and CD4+IL-17+ cells [21].

Aside from younger age, the responding group was characterised by an earlier age at which the disease was diagnosed. Interestingly, we did not observe any differences in disease duration, which were found in other studies [5]. This can be due to the non-specific onset of the disease, which is often ignored by patients at first, and in some cases, an extended period between first appearance of symptoms to the diagnosis and introduction of treatment. Symptom onset is often insufficient to determine a disease entity, even more so with the heterogeneous patient profile in AS. According to the New York criteria for AS, radiological evidence is necessary to determine the disease [6]. We considered the year in which the patient fulfilled the diagnostic criteria, marking a clinically identifiable disease. We did not focus on patients in early subclinical stages due to the difficulty in establishing a clear diagnosis.

Several studies report better response to anti-TNFα treatment in patients with higher disease activity and increased inflammatory markers [3–5, 22]. In our study, the responding group showed a tendency for higher CRP at baseline, although the difference was not significant, which could be explained by small sample size. Moreover, baseline BASDAI did not influence patient response to treatment. This could be due to the fact that differences became significant when comparing patients with baseline BASDAI 4–5 to patients with BASAI > 5 [5], whereas in our study, only one patient had baseline BASDAI below 5, while all other patients not less than 5.5.

We did not observe any differences in body mass index or BMI between the responding and non-responding groups. Although some studies report a lower response in obese patients with axial spondyloarthritis [23, 24], others showed no influence of body mass on anti-TNFα treatment in psoriatic arthritis patients [25].

Three different anti-TNFα agents were used in our study: etanercept, a recombinant receptor protein neutralising soluble TNFα; adalimumab, a human monoclonal antibody; and infliximab, a chimeric human-mouse monoclonal antibody. Both monoclonal antibodies neutralise soluble and membrane TNFα. However, response to treatment did not depend on the type of drug or route of administration. Our results are consistent with previous studies reporting that there are no differences in response between anti-TNFα agents in AS patients [4, 5, 22].

Although the small sample size is a limitation of our study, the majority of our findings are in line with previous studies performed on larger groups of patients. Therefore, our data is worth considering while planning future studies. Evaluating the response to treatment based on BASDAI improvement, despite its widespread use, can be a source of bias since it is based on patient self-assessment.

Determining cellular mechanisms involved in the clinical response to anti-TNFα drugs can allow clinicians to introduce treatment only to patients who would benefit from it and to protect others from its side effects. Besides clinical and cellular predictors of response to anti-TNFα treatment, genetic [26] and radiological [27] predictors form a new, promising approach. Accordingly, further studies defining cellular predictors of response to anti-IL-17 treatment will enable clinicians to select patients in whom anti-IL-17 agents should be considered as first-line treatment.

Our study indicates that the influence of age on the response to anti-TNFα treatment in AS patients can be mediated by TNFα-producing CD8 cells. This finding brings us closer to personalisation of therapy, which will make biological treatment more effective and accessible to a greater group of patients who would benefit from it.

## Electronic supplementary material

ESM 1 (PDF 158 kb)

ESM 2 (PDF 174 kb)

## Abbreviations

AS: ankylosing spondylitis
ASDAS: Ankylosing Spondylitis Disease Activity Score
BASDAI: Bath Ankylosing Spondylitis Disease Activity Index
CD: cluster of differentiation
CRP: C-reactive protein
csDMARDs: conventional synthetic disease-modifying antirheumatic drugs
EDTA: ethylenediaminetetraacetic acid
FBS: fetal bovine serum
FMO: Fluorescence Minus One
FSC: forward-scattered
IFN: interferon
IL: interleukin
LSM: lymphocyte separation medium
NSAIDs: non-steroidal anti-inflammatory drugs
PBMC: peripheral blood mononuclear cells
PBS: phosphate-buffered saline
RA: rheumatoid arthritis
SSC: side-scattered
TNF: tumour necrosis factor
VAS: visual analogue scale

## References

[1] van der Heijde D, Ramiro S, Landewe R, Baraliakos X, Van den Bosch F, Sepriano A, Regel A, Ciurea A, Dagfinrud H, Dougados M, van Gaalen F, Geher P, van der Horst-Bruinsma I, Inman RD, Jongkees M, Kiltz U, Kvien TK, Machado PM, Marzo-Ortega H, Molto A, Navarro-Compan V, Ozgocmen S, Pimentel-Santos FM, Reveille J, Rudwaleit M, Sieper J, Sampaio-Barros P, Wiek D, Braun J (2017). 2016 update of the ASAS-EULAR management recommendations for axial spondyloarthritis. Ann Rheum Dis.

[2] Chen JM, Veras MMS, Liu C, Lin JF (2013) Methotrexate for ankylosing spondylitis. Cochrane Db Syst Rev (2):Cd004524. doi:10.1002/14651858.Cd004524.Pub4 10.1002/14651858.CD004524.pub4 PMC11711296 23450553

[3] Rudwaleit M, Claudepierre P, Wordsworth P, Cortina EL, Sieper J, Kron M, Carcereri-de-Prati R, Kupper H, Kary S (2009). Effectiveness, safety, and predictors of good clinical response in 1250 patients treated with adalimumab for active ankylosing spondylitis. J Rheumatol.

[4] Arends S, Brouwer E, van der Veer E, Groen H, Leijsma MK, Houtman PM, Jansen TLTA, Kallenberg CGM, Spoorenberg A (2011). Baseline predictors of response and discontinuation of tumor necrosis factor-alpha blocking therapy in ankylosing spondylitis: a prospective longitudinal observational cohort study. Arthritis Res Ther.

[5] Rudwaleit M, Listing J, Brandt J, Braun J, Sieper J (2004). Prediction of a major clinical response (BASDAI 50) to tumour necrosis factor alpha blockers in ankylosing spondylitis. Ann Rheum Dis.

[6] Vanderlinden S, Valkenburg HA, Cats A (1984). Evaluation of diagnostic-criteria for ankylosing-spondylitis—a proposal for modification of the New York criteria. Arthritis Rheum.

[7] Garrett S, Jenkinson T, Kennedy LG, Whitelock H, Gaisford P, Calin A (1994). A new approach to defining disease status in ankylosing-spondylitis—the Bath Ankylosing-Spondylitis Disease-Activity Index. J Rheumatol.

[8] van der Heijde D, Lie E, Kvien TK, Sieper J, Van den Bosch F, Listing J, Braun J, Landewe R, Soc ASI (2009). ASDAS, a highly discriminatory ASAS-endorsed disease activity score in patients with ankylosing spondylitis. Ann Rheum Dis.

[9] Krabbe KS, Pedersen M, Bruunsgaard H (2004). Inflammatory mediators in the elderly. Exp Gerontol.

[10] Weyand CM, Goronzy JJ (2016). Aging of the immune system mechanisms and therapeutic targets. Ann Am Thorac Soc.

[11] Valiathan R, Ashman M, Asthana D (2016). Effects of ageing on the immune system: infants to elderly. Scand J Immunol.

[12] Zanni F, Vescovini R, Biasini C, Fagnoni F, Zanlari L, Telera A, Di Pede P, Passeri G, Pedrazzoni M, Passeri M, Franceschi C, Sansoni P (2003). Marked increase with age of type 1 cytokines within memory and effector/cytotoxic CD8(+) T cells in humans: a contribution to understand the relationship between inflammation and immunosenescence. Exp Gerontol.

[13] Duftner C, Dejaco C, Kullich W, Klauser A, Goldberger C, Falkenbach A, Schirmer M (2006). Preferential type 1 chemokine receptors and cytokine production of CDs28(−) T cells in ankylosing spondylitis. Ann Rheum Dis.

[14] Bryl E, Vallejo AN, Weyand CM, Goronzy JJ (2001). Down-regulation of CD28 expression by TNF-alpha. J Immunol.

[15] Vallejo AN, Nestel AR, Schirmer M, Weyand CM, Goronzy JJ (1998). Aging-related deficiency of CD28 expression in CD4+ T cells is associated with the loss of gene-specific nuclear factor binding activity. J Biol Chem.

[16] Duftner C, Goldberger C, Falkenbach A, Wurzner R, Falkensammer B, Pfeiffer KP, Maerker-Hermann E, Schirmer M (2003). Prevalence, clinical relevance and characterization of circulating cytotoxic CD4(+)CD28(−) T cells in ankylosing spondylitis. Arthritis Res Ther.

[17] Julia A, Erra A, Palacio C, Tomas C, Sans X, Barcelo P, Marsal S (2009). An eight-gene blood expression profile predicts the response to infliximab in rheumatoid arthritis. PLoS One.

[18] Liao HT, Lin YF, Tsai CY, Chou CT (2015). Regulatory T cells in ankylosing spondylitis and the response after adalimumab treatment. Joint Bone Spine.

[19] Toubi E, Kessel A, Mahmudov Z, Hallas K, Rozenbaum M, Rosner AI (2005). Increased spontaneous apoptosis of CD4(+)CD25(+) T cells in patients with active rheumatoid arthritis is reduced by infliximab. Ann N Y Acad Sci.

[20] Grundstrom J, Linton L, Thunberg S, Forsslund H, Janczewska I, Befrits R, van Hage M, Gafvelin G, Eberhardson M (2012). Altered immunoregulatory profile during anti-tumour necrosis factor treatment of patients with inflammatory bowel disease. Clin Exp Immunol.

[21] Li XY, Chen LN, Wu ZB, Han Q, Li Q, Ping Z (2013). Levels of circulating Th17 cells and regulatory T cells in ankylosing spondylitis patients with an inadequate response to anti-TNF-alpha therapy. J Clin Immunol.

[22] Spadaro A, Lubrano E, Marchesoni A, D'Angelo S, Ramonda R, Addimanda O, Perrotta FM, Olivieri I, Punzi L, Salvarani C (2013). Remission in ankylosing spondylitis treated with anti-TNF-alpha drugs: a national multicentre study. Rheumatology.

[23] Gremese E, Bernardi S, Bonazza S, Nowik M, Peluso G, Massara A, Tolusso B, Messuti L, Miceli MC, Zoli A, Trotta F, Govoni M, Ferraccioli G (2014). Body weight, gender and response to TNF-alpha blockers in axial spondyloarthritis. Rheumatology.

[24] Micheroli R, Hebeisen M, Wildi LM, Exer P, Tamborrini G, Bernhard J, Moller B, Zufferey P, Nissen MJ, Scherer A, Ciurea A, Qua RSC (2017). Impact of obesity on the response to tumor necrosis factor inhibitors in axial spondyloarthritis. Arthritis Res Ther.

[25] Iannone F, Fanizzi R, Scioscia C, Anelli MG, Lapadula G (2013). Body mass does not affect the remission of psoriatic arthritis patients on anti-TNF-alpha therapy. Scand J Rheumatol.

[26] Liu J, Dong Z, Zhu Q, He DY, Ma YY, Du AP, He F, Zhao DB, Xu X, Zhang H, Jin L, Wang JC (2016). TNF-alpha promoter polymorphisms predict the response to etanercept more powerfully than that to infliximab/adalimumab in spondyloarthritis. Sci Rep-Uk.

[27] Rudwaleit M, Schwarzlose S, Hilgert ES, Listing J, Braun J, Sieper J (2008). MRI in predicting a major clinical response to anti-tumour necrosis factor treatment in ankylosing spondylitis. Ann Rheum Dis.

