# Supplementary material for: Age determines response to anti-TNFα treatment in patients with ankylosing spondylitis and is related to TNFα-producing CD8 cells
Source: Clin Rheumatol. 2018 Mar 15;37(6):1597–604. doi: 10.1007/s10067-018-4061-y (PMC5948268; doi:10.1007/s10067-018-4061-y)
Supplement: Supplementary file 1 — (PDF 158 kb) [file 10067_2018_4061_MOESM1_ESM.pdf]

Technical Data Sheet

Leukocyte Activation Cocktail, with BD GolgiPlug™

Product Information

Material Number: **550583**

Size: 200 µL

Description

The most critical step for detection of intracellular accumulations of cytokines by intracellular staining is the isolation and activation of a cell population to induce production of the cytokine of interest. Without properly activated cytokine-producing cells, cultured in the presence of a protein transport inhibitor, the ability to detect intracellular accumulations of cytokines in most cases is severely compromised. Various in vitro methods have been reported for stimulating cells to produce cytokines. Polyclonal activators have been particularly useful for inducing cytokine-producing cells. These activators include the following: concanavalin A, lipopolysaccharide, phorbol esters plus calcium ionophore or ionomycin, phytohaemaglutinin, staphylococcus enterotoxin B, and monoclonal antibodies directed against subunits of the TCR/CD3 complex (with or without antibodies directed against costimulatory receptors, such as CD28).

The Leukocyte Activation Cocktail, with BD GolgiPlug™ is a ready -to-use polyclonal cell activation mixture containing the phorbol ester, PMA (Phorbol 12-Myristate 13-Acetate), a calcium ionophore (Ionomycin) and the protein transport inhibitor BD GolgiPlug™ (Brefeldin A). This mixture may be utilized to elicit a primary cytokine response from T cells. Stimulation of cells using this cocktail results in cytokine production that is localized in the rough endoplasmic reticulum of cytokine -producing cells. This localization of cytokines is caused by the protein transport inhibitor component, Brefeldin A which blocks intracellular protein transport processes. The increased accumulation of cytokine protein levels in cells thus enhances detectability of cytokine-producing cells by immunofluorescent staining and flow cytometric analysis. The contents of each 100 µL vial is sufficient for treating up to 50 mL of cell cultures (at ~10^6 cells/mL); therefore one can stimulate ≥ 5 × 10^7 cells.

Preparation and Storage

Store product at -80°C prior to use or for long term storage of stock solutions.

Application Notes

Recommended Assay Procedure:

Procedure for Using Leukocyte Activation Cocktail, with BD GolgiPlug™ : Rapidly thaw the cocktail at 37°C in a water bath and add 2 µL of cocktail for every 1 mL of cell culture (e.g., ~10^6 cells/mL) and mix thoroughly. Place culture in a 37°C humidified CO2 incubator for 4-6 hr. Following activation, harvest and wash cells with FACS Staining Buffer (e.g., BD Pharmingen™ Stain Buffer, with FBS, Cat. No. 554656) for use in antibody staining protocols. Treatment of stimulated cells for 4 to 6 hours with Leukocyte Activation Cocktail, with BD GolgiPlug significantly increases the ability to detect cytokine-producing cells by immunofluorescent staining. It is recommended that Leukocyte Activation Cocktail, with BD GolgiPlug not be kept in cell culture for longer than 12 hours. BD GolgiPlug been found to have differential effects on intracellular cytokine staining that is time, activator and cytokine dependent, therefore the need to activate cells for periods longer than 4-6 hr may be determined empirically by the investigator.

Suggested Companion Products

Catalog Number	Name	Size	Clone
554656	Stain Buffer (FBS)	500 mL	(none)

Product Notices

1. Since applications vary, each investigator should titrate the reagent to obtain optimal results.
2. Please refer to www.bdbiosciences.com/pharmingen/protocols for technical protocols.

References

Assenmacher M, Schmitz J, Radbruch A. Flow cytometric determination of cytokines in activated murine T helper lymphocytes: expression of interleukin-10 in interferon-gamma and in interleukin-4-expressing cells. *Eur J Immunol.* 1994; 24(5):1097-1101. (Biology)

Elson LH, Nutman TB, Metcalfe DD, Prussin C. Flow cytometric analysis for cytokine production identifies T helper 1, T helper 2, and T helper 0 cells within the human CD4+CD27- lymphocyte subpopulation. *J Immunol.* 1995; 154(9):4294-4301. (Biology)

Jung T, Schauer U, Heusser C, Neumann C, Rieger C. Detection of intracellular cytokines by flow cytometry. *J Immunol Methods.* 1993; 159(1-2):197-207. (Biology)

Prussin C, Metcalfe DD. Detection of intracytoplasmic cytokine using flow cytometry and directly conjugated anti-cytokine antibodies. *J Immunol Methods.* 1995; 188(1):117-128. (Biology)

Sander B, Andersson J, Andersson U. Assessment of cytokines by immunofluorescence and the paraformaldehyde-saponin procedure. *Immunol Rev.* 1991; 119:65-93. (Biology)

Vikingsson A, Pederson K, Muller D. Enumeration of IFN-gamma producing lymphocytes by flow cytometry and correlation with quantitative measurement of IFN-gamma. *J Immunol Methods.* 1994; 173(2):219-228. (Biology)

BD Biosciences

bdbiosciences.com

United States	Canada	Europe	Japan	Asia Pacific	Latin America/Caribbean
877.232.8995	866.979.9408	32.2.400.98.95	0120.8555.90	65.6861.0633	55.11.5185.9995

For country contact information, visit **bdbiosciences.com/contact**

Conditions: The information disclosed herein is not to be construed as a recommendation to use the above product in violation of any patents. BD Biosciences will not be held responsible for patent infringement or other violations that may occur with the use of our products. Purchase does not include or carry any right to resell or transfer this product either as a stand-alone product or as a component of another product. Any use of this product other than the permitted use without the express written authorization of Becton, Dickinson and Company is stictly prohibited.

For Research Use Only. Not for use in diagnostic or therapeutic procedures. Not for resale.

Unless otherwise noted, BD, BD Logo and all other trademarks are property of Becton, Dickinson and Company. © 2015 BD
