# Supplementary material for: Age determines response to anti-TNFα treatment in patients with ankylosing spondylitis and is related to TNFα-producing CD8 cells
Source: Clin Rheumatol. 2018 Mar 15;37(6):1597–604. doi: 10.1007/s10067-018-4061-y (PMC5948268; doi:10.1007/s10067-018-4061-y)
Supplement: Supplementary file 2 — (PDF 174 kb) [file 10067_2018_4061_MOESM2_ESM.pdf]

Technical Data Sheet

Fixation and Permeabilization Solution

Product Information

Material Number: **554722**
Size: 125 mL

Description

BD Cytofix/Cytoperm™ solution is supplied as a 1X solution and can be used for the simultaneous fixation and permeabilization of cells prior to intracellular cytokine staining.

Preparation and Storage

Store undiluted at 4°C.

Application Notes

Application

Intracellular staining (flow cytometry)	Routinely Tested

Recommended Assay Procedure:

For the stimulation of cells, various *in vitro* methods have been reported for stimulating cells to produce cytokines. Polyclonal activators have been particularly useful for inducing cytokine-producing cells. These activators include the following: concanavalin A, lipopolysaccharide, phorbol esters plus calcium ionophore or ionomycin, phytohaemaglutinin, staphylococcus enterotoxin B, and monoclonal antibodies directed against subunits of the TCR/CD3 complex (with or without antibodies directed against costimulatory receptors, such as CD28).

1. Fix and Permeabilize Cells

a. Thoroughly resuspend cells in 100 µL of BD Cytofix/Cytoperm solution per well for microwell plates (or 250 µL for tubes) and incubate for 20 min. at 4°C. ***Note:*** Cell aggregation can be avoided by vortexing prior to the addition of the BD Cytofix/Cytoperm™ solution.
b. Wash cells two times in a buffer that contains a cell permeabilizing agent such as saponin (BD Perm/Wash™ buffer, Cat. 554723, which can be used as the wash buffer and as the antibody diluent).

2. Stain for Intracellular Cytokines

a. Thoroughly resuspend fixed/permeabilized cells in 50 µL of a saponin-containing buffer (e.g., BD Perm/Wash™ buffer) containing a pre-determined optimal concentration of a fluorochrome-conjugated anti-cytokine antibody or appropriate negative control. Incubate at 4°C for 30 minutes in the dark. ***Note:*** Because saponin-mediated cell permeabilization is a reversible process, it is important to keep the cells in the presence of saponin during intracellular cytokine staining.
b. Wash cells 2 times with saponin-containing buffer (e.g, BD Perm/Wash™ buffer) and resuspend in staining buffer prior to flow cytometric analysis.

Note: Both the BD Cytofix/Cytoperm™ (Cat. No. 554722) and BD Perm/Wash buffer (Cat. No. 554723) are included in the Fixation/Permeabilization Solution Kit (Cat. No. 554714) as well as the Fixation/Permeabilization Solution Kit with BD GolgiStop (containing monensin); Cat. No. 554715) and Fixation/Permeabilization Solution Kit with GolgiPlug (containing brefeldin A); Cat. No. 555028).

BD Biosciences
bdbiosciences.com

United States	Canada	Europe	Japan	Asia Pacific	Latin America/Caribbean
877.232.8995	866.979.9408	32.2.400.98.95	0120.8555.90	65.6861.0633	55.11.5185.9995

For country contact information, visit **bdbiosciences.com/contact**

Conditions: The information disclosed herein is not to be construed as a recommendation to use the above product in violation of any patents. BD Biosciences will not be held responsible for patent infringement or other violations that may occur with the use of our products. Purchase does not include or carry any right to resell or transfer this product either as a stand-alone product or as a component of another product. Any use of this product other than the permitted use without the express written authorization of Becton, Dickinson and Company is stictly prohibited.
For Research Use Only. Not for use in diagnostic or therapeutic procedures. Not for resale.
Unless otherwise noted, BD, BD Logo and all other trademarks are property of Becton, Dickinson and Company. © 2015 BD

Danger
BD Cytofix/Cytoperm™ Buffer (Fixation and Permeabilization Solution) contains 4.2% formaldehyde (w/w).

Hazard statements
Harmful if inhaled.
Causes skin irritation.
Causes serious eye damage.
May cause an allergic skin reaction.
Suspected of causing genetic defects.
May cause cancer. Route of exposure: Inhalative.
May cause respiratory irritation.

Precautionary statements
Wear protective clothing / eye protection.
Wear protective gloves.
Do not breathe mist/vapours/spray.
IF IN EYES: Rinse cautiously with water for several minutes. Remove contact lenses, if present and easy to do.
Continue rinsing.
If skin irritation or rash occurs: Get medical advice/attention.

Suggested Companion Products

Catalog Number	Name	Size	Clone
554723	Perm/Wash Buffer	100 mL	(none)
554714	BD Cytofix/Cytoperm™ Fixation/Permeablization Kit	250 Tests	(none)
554715	BD Cytofix/Cytoperm Plus Kit (with BD GolgiStop)	250 Tests	(none)
555028	BD Cytofix/Cytoperm Plus Kit (with BD GolgiPlug)	250 Tests	(none)

Product Notices

1. Please refer to www.bdbiosciences.com/pharmingen/protocols for technical protocols.

References

Assenmacher M, Schmitz J, Radbruch A. Flow cytometric determination of cytokines in activated murine T helper lymphocytes: expression of interleukin-10 in interferon-gamma and in interleukin-4-expressing cells. *Eur J Immunol.* 1994; 24(5):1097-1101. (Methodology)
Elson LH, Nutman TB, Metcalfe DD, Prussin C. Flow cytometric analysis for cytokine production identifies T helper 1, T helper 2, and T helper 0 cells within the human CD4+CD27- lymphocyte subpopulation. *J Immunol.* 1995; 154(9):4294-4301. (Methodology)
Jung T, Schauer U, Heusser C, Neumann C, Rieger C. Detection of intracellular cytokines by flow cytometry. *J Immunol Methods.* 1993; 159(1-2):197-207. (Methodology)
Prussin C, Metcalfe DD. Detection of intracytoplasmic cytokine using flow cytometry and directly conjugated anti-cytokine antibodies. *J Immunol Methods.* 1995; 188(1):117-128. (Methodology)
Sander B, Andersson J, Andersson U. Assessment of cytokines by immunofluorescence and the paraformaldehyde-saponin procedure. *Immunol Rev.* 1991; 119:65-93. (Methodology)

BD Biosciences
bdbiosciences.com

United States	Canada	Europe	Japan	Asia Pacific	Latin America/Caribbean
877.232.8995	866.979.9408	32.2.400.98.95	0120.8555.90	65.6861.0633	55.11.5185.9995

For country contact information, visit **bdbiosciences.com/contact**

Conditions: The information disclosed herein is not to be construed as a recommendation to use the above product in violation of any patents. BD Biosciences will not be held responsible for patent infringement or other violations that may occur with the use of our products. Purchase does not include or carry any right to resell or transfer this product either as a stand-alone product or as a component of another product. Any use of this product other than the permitted use without the express written authorization of Becton, Dickinson and Company is stictly prohibited.
For Research Use Only. Not for use in diagnostic or therapeutic procedures. Not for resale.
Unless otherwise noted, BD, BD Logo and all other trademarks are property of Becton, Dickinson and Company. © 2015 BD
